# Health services uptake among nomadic pastoralist populations in Africa: A systematic review of the literature

**DOI:** 10.1371/journal.pntd.0008474

**Published:** 2020-07-27

**Authors:** Victoria M. Gammino, Michael R. Diaz, Sarah W. Pallas, Abigail R. Greenleaf, Molly R. Kurnit

**Affiliations:** 1 Global Immunization Division, Center for Global Health, US Centers for Disease Control and Prevention. Atlanta, Georgia, United States of America; 2 Department of Population, Family and Reproductive Health, Bloomberg School of Public Health, Johns Hopkins University, Baltimore, Maryland, United States of America; Universidade do Estado do Rio de Janeiro, BRAZIL

## Abstract

The estimated 50 million nomadic pastoralists in Africa are among the most “hard-to-reach” populations for health-service delivery. While data are limited, some studies have identified these communities as potential disease reservoirs relevant to neglected tropical disease programs, particularly those slated for elimination and eradication. Although previous literature has emphasized the role of these populations’ mobility, the full range of factors influencing health service utilization has not been examined systematically. We systematically reviewed empirical literature on health services uptake among African nomadic pastoralists from seven online journal databases. Papers meeting inclusion criteria were reviewed using STROBE- and PRISMA-derived guidelines. Study characteristics were summarized quantitatively, and 10 key themes were identified through inductive qualitative coding. One-hundred two papers published between 1974–2019 presenting data from 16 African countries met our inclusion criteria. Among the indicators of study-reporting quality, limitations (37%) and data analysis were most frequently omitted (18%). We identified supply- and demand-side influences on health services uptake that related to geographic access (79%); service quality (90%); disease-specific knowledge and awareness of health services (59%); patient costs (35%); contextual tailoring of interventions (75%); social structure and gender (50%); subjects’ beliefs, behaviors, and attitudes (43%); political will (14%); social, political, and armed conflict (30%); and community agency (10%). A range of context-specific factors beyond distance to facilities or population mobility affects health service uptake. Approaches tailored to the nomadic pastoralist lifeway, e.g., that integrated human and veterinary health service delivery (a.k.a., “One Health”) and initiatives that engaged communities in program design to address social structures were especially promising. Better causal theorization, transdisciplinary and participatory research methods, clearer operational definitions and improved measurement of nomadic pastoralism, and key factors influencing uptake, will improve our understanding of how to increase accessibility, acceptability, quality and equity of health services to nomadic pastoralist populations.

## Author summary

There are approximately 50 million nomadic pastoralists in Africa for whom there is little data on healthcare access and utilization. This data scarcity presents a challenge to prevent, treat and control neglected tropical diseases and design the health service delivery mechanisms through which these objectives can be met. Examining a range of studies conducted over a 45-year period, we identified supply- and demand-side influences on health services uptake in ten thematic areas. These included physical proximity to, and quality of, health services; monetary and opportunity costs of accessing care; and societal and gender norms governing power dynamics within nomadic pastoralist groups as well as those between them and health care providers. The knowledge, attitudes and practices of health care providers and health seekers also played a role in utilization, as did hegemonic factors including “political will” and varying degrees of social conflict. NTD research topics included guinea worm, lymphatic filariasis, rabies, soil-transmitted helminths, tuberculosis (bovine and human), cholera, and rift valley fever. Studies pertaining to community-directed initiatives and “One Health” approaches offered promising solutions to increase service uptake. We recommend ways to strengthen future research on this subject to improve health service delivery to, and uptake among, nomadic pastoralist populations.

## Introduction

### Background and rationale

The World Health Organization estimates that over 600 million people in Africa are impacted by Neglected Tropical Diseases (NTDs)[1–3]. And within Africa, nomadic pastoralists remain among those African populations with higher rates of morbidity and mortality for a number of preventable conditions [4–6]. Nomadic pastoralists have been of particular concern since the smallpox eradication program era in the late 1970s, the success of which relied on high levels of herd immunity [7]. Contemporary elimination and eradication programs in Africa (e.g., tuberculosis, polio and dracunculiasis) additionally observe that individuals moving between localities may serve as potential disease reservoirs or vectors [4, 8–11]. Multidisciplinary efforts to optimize the health of humans, animals, and the environment (a.k.a. “One Health” initiatives) have also demonstrated that these populations may be systematically missed by formal health care systems, owing to their mobility and systemic factors [12–15]. The demographic invisibility of nomadic pastoralist populations is particularly salient in light of the Sustainable Development Goal (SDG) 2030 targets which require accurate denominator data to assess whether indicators are reached [16].

While cultural and logistical barriers to formal, allopathic health service uptake by nomadic pastoralists have been recognized in the published literature since at least 1928, operationalizing a definition of nomadic pastoralism that is both valid and robust enough for research purposes has proven elusive and has hindered research efforts [17]. Nomadism typically refers to the lifestyle of mobile communities lacking a fixed residence while pastoralism refers to the raising of livestock; nomadic pastoralism is a subset at the intersection of the broader categories of nomads and pastoralists, which emerges out of specific ecological, cultural, political, and economic conditions. Pastoral production (i.e., livestock raising) is an adaptation to arid and semi-arid conditions, and mobility is, in turn, an often necessary response to the dynamic environmental conditions that influence the water and grazing resources critical to successful production. Although the cultures and social structures of nomadic pastoralist societies vary widely, they generally share subsistence patterns that revolve around communal land use. In Africa, agricultural livelihoods are rarely either purely pastoral or purely crop cultivation; they are more commonly a blend of the two, the continuum of which is shaped by ecosystem and culture [18]. Given this continuum, as well as localized ethnic and cultural differences, the terms nomadism, semi-nomadism, pastoralism, and mixed-methods livelihoods are frequently conflated by researchers, resulting in systematic measurement error in research related to nomadic pastoralism that impedes programmatic efforts as well as the trajectory of scientific inquiry in this area [19].

Synthesis of research findings is complicated not only by definitional discrepancies but also because the existing literature spans multiple disciplines. While research about nomadic pastoralism is more common among disciplines such as anthropology, sociology, and the veterinary and environmental sciences, many of the studies that focus specifically on health services uptake among these populations have been conducted using biomedical approaches typical of medicine and epidemiology [10]. Owing to differences in epistemology and methods, much of the biomedical literature features an outsider (or etic) perspective that seeks to identify objective factors contributing to population health, versus the insider (or emic) perspectives more typical in social science disciplines that seek to document the internal logic of nomadic pastoralists’ beliefs, behaviors, and social structures. The biomedical literature has thus contributed to a “conventional wisdom” around the reasons for low uptake of formal sector health services among nomadic pastoralists focused on a limited set of factors that are often incompletely theorized or empirically measured. The most common of these tropes relate to “long distances” (an assumption based on itinerancy) and narratives about lengthy and unpredictable migration patterns; the role of “cultural differences” and “poverty” in health-seeking behaviors and access to care; and “education level” (or its surrogate, literacy) affecting demand for services [20, 21]. When these tropes persist in the absence of valid operational definitions and empirical measures that also address correlations and confounding, or in spite of empirical data to the contrary, there is a risk of drawing inaccurate conclusions and proposing ineffective solutions to increase nomadic pastoralists’ health service uptake.

Two previous peer-reviewed syntheses of this topic have been conducted, however neither were systematic [22, 23]. To address this gap, we conducted a systematic review of determinants of health services uptake among nomadic pastoralists in Africa. We focused our review on Africa as a geography in which there are numerous nomadic pastoralist communities and in which “demographic invisibility” and low health service uptake among these communities has been identified as a concern for current disease eradication and elimination efforts and achievement of the SDGs [24]. On the basis of our results synthesis, we identify potential strategies to increase health services uptake and directions for future research.

## Methods

### Literature search strategy

We conducted a systematic search of literature indexed in seven online journal databases using search terms related to health services uptake, nomadic, transhumant, or itinerant pastoralists, and limited to locations in continental Africa published on or before February 28, 2019 (S1 Appendix). The databases searched were Medline (OVID), Embase (OVID), Global Health (OVID), PsycINFO (OVID), Sociological Abstracts (ProQuest Central), Scopus, and African Index Medicus. The search terms were intentionally broad due to the lack of standardized language or medical subject heading (MeSH) terms related to nomadic pastoralists. We used no language or date restrictions in the search.

### Selection of studies

The initial search identified 5647 papers, which was reduced to 4354 papers after removing duplicates (S1 Fig). Of those papers, only the 833 titles and abstracts that included one or more of the keywords “nomadic”, “transhumant”, "itinerant” and/or “pastoralist” (and relevant variants) were evaluated for topical relevance by two reviewers (VMG, MRD) to determine if they warranted full-text review. Conference abstracts, book chapters, papers that were not peer-reviewed (i.e., “gray literature”), papers that were clinically rather than population-health focused (e.g., genetics studies), or papers in which the sole study population was sedentary or non-human were excluded. On the basis of this assessment, a total of 195 full-text papers were retained for full review. The final review set included all original peer-reviewed papers that (1) focused on the implementation or evaluation of an intervention to increase health services uptake or (2) reported a study that identified, characterized, or assessed opportunities or strategies related to health services uptake in our target population. Given the dearth of research on nomadic pastoralists (most notably of experimental studies), we placed no restrictions on study design. A total of 102 papers met the inclusion criteria and were reviewed.

### Paper review

All reviews were conducted using a standardized, piloted data abstraction form comprising 44 questions. Data were entered using the SurveyMonkey platform. Each paper was reviewed by two independent reviewers with discordant answers resolved by consensus. Papers written in French were reviewed by French-speaking reviewers (SWP, ARG). To account for the range of study types, we derived the indicators of study reporting-quality contained in our abstraction instrument based on the Strengthening the Reporting of Observational Studies in Epidemiology (STROBE) statement for reporting observational studies [25]. In addition to the STROBE-derived elements (Table 1), the abstraction form included additional open-ended questions informed by Preferred Reporting Items for Systematic Reviews and Meta-Analyses (PRISMA) review guidelines regarding study characteristics including: (i) geographic and study population details; (ii) research question/objectives, study design, sample size, and potential sources of bias; (iii) facilitators and barriers to service uptake; and (iv) summaries of proposed interventions, strategies and recommendations related to increasing health service uptake [S1 PRISMA checklist, 26]. We classified study designs using Rothman and Greenland’s definitions [27].

**Table 1:** Study reporting quality indicators.

Reporting quality indicator (N = 102)	No. (%)
Research question/hypothesis/objectives clearly stated	98 (96)
Description of the target population present	98 (96)
Participant recruitment or sampling methodology described	100 (98)
Methods section present	97 (95)
Data analysis described	84 (82)
Results presented	102 (100)
Findings compared to similar studies	87 (85)
Limitations noted	64 (63)
Conclusions presented	102 (100)

### Data Analysis

Abstracted data were exported from SurveyMonkey into Microsoft Excel 2016 for data management and cleaning. Descriptive quantitative analyses were conducted using IBM SPSS ver. 25 software. The authors reviewed responses from the open-ended questions pertaining to facilitators, barriers and strategies relating to uptake to identify salient, crosscutting themes using an iterative inductive coding method [28, 29].

## Results

### General characteristics of papers

#### Geographic locations covered

Studies were conducted in 16 African countries, with the highest number of studies from Ethiopia (33), Kenya (17), Chad (13), Nigeria (12), and Tanzania (7). Ninety-eight percent of papers designated a specific geographic region within a country for the study.

#### Nomadic pastoralist population characteristics

Within our sample, four principal labels were used for the study populations: “nomadic” (33), “pastoralist” (33), “semi-nomadic pastoralist” (17), “nomadic-pastoralist” (19); five variants on these terms had a frequency less than 10, with some papers using multiple terms. In 53 (52%) papers, the pastoralists’ movement patterns were characterized as: (i) nomadic (21): demonstrating non-fixed migration patterns; (ii) transhumant (12): demonstrating consistent or fixed seasonal migration patterns along well-defined routes; and (iii) semi-nomadic/semi-sedentary (20): living in sedentary settlements part of the year. Sixty-one papers (60%) did not specify the livestock type raised by the study subjects although grazing and associated human migration patterns vary significantly by species. Among those that did, the principal livestock raised included cattle (30%) and camels (15%); 24% of papers indicated that more than one species was cultivated.

#### Sample sizes and time periods covered

Given the variability in subjects and methods, the sample size of studies ranged from less than ten for a qualitative assessment of traditional beliefs, to a high of approximately 96,376 nomads verbally screened for active tuberculosis (TB) [30, 31]. The included papers were published between 1974 and 2019, and most were published after 2013, consistent with trends in the increasing volume of scientific publications generally [32, S2 Fig].

### Study designs, quality of study reporting, and risks of bias

#### Study designs

The majority of research activities were non-experimental in nature; the most common non-experimental study designs were cross-sectional surveys (58) and qualitative studies (45). Of the 15 studies employing an experimental design, there were 11 community interventions and four clinical or field trials. Thirty-four studies (33%) used more than one method. The remaining study types (e.g., cohort or case studies) had a frequency of two or less.

#### Quality of study reporting

Of the 102 studies that met the inclusion criteria, 99 (97%) of the papers contained six or more indicators of study-reporting quality (Table 1). Eighty-five (83%) described how data were analyzed, 87 (85%) compared findings to other studies, and 64 (63%) noted study limitations. Most studies followed a standard reporting format, although only 84 (83%) of papers had both a methods section and described the data analysis, and only 60 (59%) of papers reported both the limitations and compared their findings to other studies. While all papers offered conclusions, 11 offered no recommendations and 10 others lacked specific or actionable recommendations, instead making broad suggestions such as “improve access to care” or “implement proper policy measures.”

#### Assessment and description of potential bias

Reviewers identified one or more potential sources of bias in 45 (44%) papers. Among these, selection (36), confounding (17), and reporting biases (8) were identified as the most common potential problems.

### Research areas and study objectives

#### Research areas

Five broad areas of research were identified among the included papers. The first area included epidemiologic studies that focused primarily on the prevalence of, and biomedical risk factors for, specific diseases or conditions, such as cholera, TB, and rabies (16). A second area comprised studies focused on the knowledge, attitudes and practices specific to certain conditions (e.g., pregnancy) and diseases (e.g., Rift Valley Fever) and the factors determining or correlating with knowledge, attitudes and practices (25). A third category focused on operational research intended to improve diagnosis and management of specific diseases (e.g., rabies), conditions (e.g., home delivery for pregnant women), technologies (e.g., biometric registration systems), or the feasibility of specific approaches (e.g., community-directed interventions, integrated human-veterinary services known as “One Health”) (n = 50). The fourth area included formative and other qualitative research to develop hypotheses or guide the design of interventions (n = 6). A final research area addressed policy and practice issues pertaining to perceived barriers, for example by evaluating the role of cash-based funds from Gavi, the Vaccine Alliance; task-shifting to counter human resource shortages in remote under-served areas; or the acceptance of private, non-employer-supported insurance policies for the poor (n = 5).

#### Specific human health topics

The health topic foci included reproductive health issues (including antenatal care, maternal mortality and/or childbirth) (21); vaccine-preventable disease/immunization topics (excluding polio) (14); general health service access, uptake or delivery (9); primary health care (4); or disease-specific topics such as tuberculosis (16), neglected tropical diseases (9), polio (9), malaria (8), HIV/AIDS (4) and zoonoses (3) (S2 Fig). Five papers on other unique topics were also reviewed (not listed individually).

#### Themes impacting health services uptake by nomadic pastoralists

Ten crosscutting themes emerged from the iterative, inductive coding of the extracted data. Where indicated, sub-themes are also reported regarding facilitators of, barriers to and recommendations for improving the uptake of services (studies categorized under each theme are reported in Table 2; some studies had multiple sub-themes).

**Table 2:** Final included literature coding by theme of barriers to, facilitators of, and recommendations for improving uptake of health services (N = 102 articles).

Theme	Barriers	Facilitators	Recommendations
**1. Distance/ geographic access (n = 81)**	[4, 5, 9, 20, 31, 33–93]	[4, 31, 35, 38, 40, 41, 44, 47, 48, 50, 53, 62, 65, 67–72, 76, 78, 79, 82, 83, 88, 90, 94–96]	[9, 20, 30, 31, 33–35, 37–41, 43, 44, 46–56, 58, 60, 63, 66, 67, 69, 72, 74, 83, 91–94, 97–107]
**2. Health service quality (n = 92)**	[4, 9, 30, 33, 35, 36, 38, 39, 42–46, 48, 49, 51, 52, 54, 55, 57, 58, 60–63, 68, 70, 74, 76, 77, 79–87, 89–92, 94, 98, 103, 104, 107–117]	[4, 9, 31, 33, 35, 38, 40, 41, 47, 53, 54, 58, 60, 65, 66, 71, 74–76, 79, 80, 82, 85, 88–91, 93, 97–99, 101, 103, 104, 106, 108, 109, 112, 115, 117–120]	[4, 9, 20, 30, 34, 35, 39, 40, 42, 43, 45–50, 52–58, 60, 62–64, 66, 67, 69, 70, 72–74, 77, 79–89, 91–95, 98, 99, 101–108, 110–112, 114–124]
**3. Knowledge of disease and awareness of health services** **(n = 60)**	[5, 9, 34, 36, 37, 40, 43, 46–48, 55, 58, 62, 72, 76, 77, 79, 80, 83, 87, 88, 90, 91, 101, 102, 106, 107, 110, 112, 116, 118–122, 124–126]	[4, 9, 31, 36, 37, 43, 52, 55, 57, 62, 64, 66, 70–72, 74, 76, 80, 82, 83, 90, 91, 100, 102, 106, 108, 112, 119, 121, 122, 125, 126]	[4, 34, 36, 37, 39, 43, 46, 48–50, 52, 53, 55, 57, 62–64, 70, 77, 78, 83, 91, 95, 102, 105, 112, 115, 116, 118, 120, 122–126]
**4. Cost to nomadic pastoralists (n = 36)**	[42–44, 46, 48, 62–65, 68, 69, 71, 76, 79, 82, 83, 85, 88, 90, 110, 114, 116, 119]	[43, 55, 63, 69, 71, 79, 82, 83, 85, 90, 95, 111, 113]	[34, 35, 40, 46–48, 83, 85, 89, 90, 92, 93, 98, 99, 110, 111, 116, 119]
**5. Tailoring interventions and materials to the local context (n = 77)**	[4, 30, 59, 61, 79, 80, 83, 94, 95, 99, 100, 114, 116, 123]	[5, 20, 30, 31, 33–35, 38, 40, 41, 44, 45, 47, 49, 51, 52, 55, 57, 59, 61, 63–68, 70, 72, 73, 76–85, 91–97, 99–101, 103, 104, 109–112, 114, 117, 124, 127, 128]	[9, 20, 30, 31, 34–36, 44, 46, 54–56, 58, 59, 63, 66, 70, 74, 75, 79, 81, 83, 85, 87, 93, 95, 104–106, 109, 112, 116, 118, 119, 125]
**6. Social structure and gender (n = 51)**	[35, 36, 42, 44, 45, 55, 59, 62, 66–71, 75, 77, 79, 80, 82, 85, 90–92, 97, 99, 116, 119, 121, 123]	[30, 42, 45, 52, 61, 62, 65, 69, 79, 85]	[30, 38, 40, 42, 48, 49, 53, 55, 57, 58, 62, 64, 66, 70, 71, 78–80, 85, 88, 91–95, 97, 99, 104, 106, 109, 112, 116, 119, 120]
**7. Nomadic pastoralist beliefs, behaviors and attitudes (n = 44)**	[30, 31, 34, 35, 37, 43, 45–48, 54, 55, 57, 58, 64, 66, 68, 70, 72, 74, 76, 80, 81, 83, 85, 90, 92, 96, 97, 102, 104, 107, 108, 112, 113, 116, 118, 120, 123, 126, 128]		[30, 57, 64, 70, 71, 83, 85, 88, 92, 112, 116, 122, 126]
**8. Political will (n = 17)**	[33, 98]	[49, 92, 104]	[30, 52, 55, 60, 69, 72, 83, 85, 92, 93, 98, 104, 106, 109, 115]
**9. Social, political and armed conflict (n = 31)**	[31, 37, 42, 47, 50–52, 61, 63, 68, 69, 76–78, 82, 92, 97, 101, 114, 127, 128]		[9, 30, 38, 42, 47, 50, 52, 55, 59, 60, 69, 82, 84, 93, 97, 101, 104, 119, 127]
**10. Community agency (n = 11)**		[61, 111, 127]	[51, 52, 55, 61, 79, 85, 97–99, 127]

#### Theme: Distance/geographic access

Geographic factors were identified as barriers to the uptake of health services among nomadic pastoralists in 66 (65%) papers. These included the distance (and road conditions) between temporary camps and health facilities (45); limited operational capacity to provide outreach services to nomadic groups and difficulty locating them (19); and the inherent difficulty providing services that require multiple visits (e.g., TB treatment and multi-dose vaccines) in the absence of systems that would facilitate clinical continuity from location to location (29). There was not consensus on the irregularity of migrations however, as one paper tied their perceived regularity to the feasibility of outreach efforts. Similarly, 29 (28%) papers mentioned nomadic pastoralists' proximity to facilities, health care workers’ (HCWs) familiarity with nomadic pastoralists’ seasonal movement or knowledge of temporary camp locations (5) and adjusting delivery strategies for seasonal conditions (6), as drivers of health service uptake. Approximately 10% of papers citing distance or mobility as a factor related to access measured actual distances to health facilities or services; the remainder did not quantify distance, or reported the distance perceived or estimated by respondents. Strategies to mitigate the impact of distance and mobility specifically were proposed in 49 (48%) papers. The provision of mobile health services was mentioned in 31 papers in response to the effect of (perceived and actual) geographic distance on health service access at permanent (i.e., fixed location) health facilities. Recommendations to implement programmatic solutions (e.g., modify TB regimens, adopt flexible catch-up vaccination schedules) were made in 25 (25%) papers.

#### Theme: Health service quality

The poor quality of health services delivered to nomadic pastoralists was identified as a barrier to uptake in 58 (57%) papers. Structural factors impacting the quality of services included deficient infrastructure, equipment, supplies, and health products (e.g., vaccines and medicines) (35); insufficient or poor-quality data (e.g., for logistical planning) (9); and inadequate numbers of appropriately trained health care personnel (30). The behavior and attitudes of formal sector HCWs (e.g. rudeness and prejudice) toward patients were identified in 16 and 4 papers respectively. By comparison, 43 (42%) papers found that high-performing service delivery facilitated access. Well-trained HCWs (12), effective, targeted health communications (19), service reliability and stability of health supplies (12), or community members’ positive perceptions of the quality and value of services (21) were specifically identified as drivers of health service uptake. Explicit recommendations to improve the quality of health services were found in 75 (74%) papers. These included structural improvements in equipment, supplies and infrastructure (37), increased training and supervision to improve HCW attitudes and behaviors towards nomadic pastoralists, and to strengthen technical and preventive services and care delivery (24). Recruitment and training of community health workers (CHWs) and traditional birth attendants was also recommended as a means to decentralize and thereby increase access to certain services (31). An important subgroup of recommendations proposed integrating human health services with those of other sectors, e.g., the veterinary and education sectors which also provide outreach to nomadic pastoralist groups (19). Nineteen papers highlighted efforts to use veterinary extension workers to conduct human disease surveillance or vaccination campaigns while four others pointed to established educational programs as a potential opportunity. Improving core public health functions, such as data for program planning, surveillance, and monitoring and evaluation, was another sub-theme (11).

#### Theme: Knowledge of disease and awareness of health services

Researchers identified nomadic pastoralists’ limited knowledge of specific diseases (25), including due to lack of education more generally (11) and limited familiarity with, and value of, formal health services to prevent and treat disease (21), as barriers to health services uptake in 38 (37%) of papers. Consistent with these findings, nomadic pastoralists’ understanding of specific diseases (including risk factors, consequences, and prevention methods) (21), and awareness of health services offered locally (21) were found to facilitate health service utilization. Recommendations to improve nomadic pastoralists’ knowledge of disease and available health services were made in 35 (34%) papers. These were inclusive of broader scopes of work for CHWs, fixed-post health workers, and veterinary extension workers to deliver education sessions in nomadic pastoralist camps, as well as mass information campaigns by radio.

#### Theme: Costs to nomadic pastoralists

Health service-associated costs were identified as a barrier to uptake in 23 (23%) papers. These costs included direct medical (e.g., out-of-pocket payments health services, informal fees at health facilities, medications), non-medical costs (transport to and from health facilities, paying someone else to shepherd while seeking care, paying for water point access near health facilities), and the indirect (or opportunity) costs of time and domestic productivity losses due to care-seeking (e.g., travel and waiting time at health facilities). Affordability of health services, such as through reduced prices or introduction of material incentive programs that offset care-seeking costs, was mentioned as facilitating health service uptake in 13 (13%) papers. Recommendations to reduce costs to nomadic pastoralists were made in 18 (18%) papers. These included providing incentives and supply-side interventions to reduce out-of-pocket and indirect costs of time required to seek care (e.g., locating health services along migration routes at seasonally appropriate times).

#### Theme: Tailoring interventions and materials to the local context

The failure to contextualize intervention designs through substantive community engagement was mentioned as a barrier to health services uptake in 14 (14%) papers. Nine papers specifically spoke to the lack of cultural relevancy. By comparison, 60 (59%) papers described the appropriate tailoring of interventions and strategies to the local context as facilitating uptake. The types of approaches credited with success generally engaged the community and local leadership (24), were adapted to the local context (43), offered flexible program logistics (25), or integrated human and animal service delivery (12). Recommendations to tailor interventions and strategies to the nomadic pastoralist context to improve the health services uptake were made in 35 papers (34%). Recommendations included season-appropriate and venue-specific delivery (e.g., marketplaces) (20), developing culturally and language-appropriate materials (17) and targeting the appropriate communications medium (e.g., radio) (6).

#### Theme: Social structure and gender

Recognizing the heterogeneity of nomadic pastoralist ethnic groups in Africa, the respective roles of societal structures (e.g., the agency associated with age, gender, marital or social status) emerged clearly as a theme. Some element of nomadic pastoralist “social structure” was identified as a barrier to formal health service uptake in 29 (28%) papers. Of these, 19 (19%) identified gender norms that impede health care access, e.g., the time demands of childcare, or those that prevent women from unaccompanied travel. Ten (10%) papers described how social structure could hypothetically drive uptake of services, for example through explicit recognition of family composition and clan hierarchy. Recommendations to adapt approaches to fit within normative social structures were found in 27 (26%) papers. Community-designed initiatives (18), efforts to explicitly recognize the role of religious and traditional leadership (13) and gender-specific considerations related to service delivery (11) were mentioned.

#### Theme: Nomadic pastoralist beliefs, behaviors and attitudes

In 41 (40%) papers, the beliefs, behaviors and attitudes ascribed to the particular nomadic pastoralists studied were identified as barriers to the uptake of formal sector health services offered at fixed-post health facilities or through outreach. Examples included nomadic pastoralists’ reported preferences for self-treatment and traditional medicine/healers (7). Studies did not always clarify, however, if this preference may have been related to factors such as cost, distance, knowledge, or quality/efficacy of formal sector health services, misconceptions about or perceived risks of “Western medicine” (e.g., rumors about vaccine side effects), or suspicions around mass outreach activities (e.g., that health gatherings are a ploy to enable taxation). The stigma associated with specific diagnoses was also cited as having a negative impact on health-seeking behavior. Recommendations to “correct” or align nomadic pastoralists’ health beliefs, behaviors and attitudes with western perspectives by increasing biomedical knowledge of disease were found in 13 (13%) papers. These strategies included engaging with religious leaders, creating formal collaborations between professional HCWs and traditional healers and birth attendants, and conducting community health education sessions.

#### Theme: Political will

Two (2%) papers explicitly mentioned issues with sustainability and insufficient political will as barriers to health service uptake among nomadic pastoralists (in Somalia and Nigeria), while three (3%) papers identified political commitment–demonstrated through government engagement and financial resource allocation–as a facilitator of uptake (in Somalia and Chad). In 15 (15%) papers, recommendations were made to increase political commitment through measures such as ministry-level coordination and collaboration (especially multisectoral collaboration among ministries of health, livestock, and agriculture) (13), sustained financial support for interventions to improve health among nomadic pastoralists (4), and improved partnerships among researchers, policy makers, and communities (2).

#### Theme: Social, political and armed conflict

The broader context of conflict within which health services may be delivered to nomadic pastoralists was noted as a barrier in 21 (21%) papers. These conflicts comprised those between nomadic and settled communities (7), nomadic pastoralists’ conflicts with government authorities/services (6), and macro-level political and economic factors of resource scarcity, insecurity, or civil conflict in the country (10). Recommendations to address such conflicts were made in 19 (19%) papers. These included implementing participatory approaches to engage nomadic pastoralist communities in health program design to: dialogue with settled communities and government around land and water resources (8); increase involvement of trusted community members (e.g., traditional leaders, healers, birth attendants) to reduce tensions (4); and separate health services from contentious government activities (e.g., taxation) while integrating with well-received non-health services (e.g., education) (9). Recommendations were mixed as to whether health service delivery approaches designed primarily for sedentary populations should be adapted to better serve nomadic pastoralists, or whether to design interventions specifically for nomadic pastoralists.

#### Theme: Community agency

The absence of community agency–generally characterized as the capacity for self-actualization demonstrated through the exertion of power–was not mentioned explicitly as a barrier to health service uptake in any of the papers reviewed. However, it was mentioned as a facilitator of health services uptake in 3 (3%) papers. Although there is scant literature describing interventions centered around community agency, 10 (10%) papers recommended that community-driven interventions and initiatives could increase health service uptake.

## Discussion

As the first systematic literature review focusing on the uptake of formal health services among nomadic pastoralists in Africa, the results highlight several previously unrecognized trends in the literature. Our review underscores the need for future research on this population and presents opportunities to address the gaps and methodological challenges identified in order to increase the evidence base for actionable, policy-relevant recommendations.

### Methodological issues of included studies

Our results reflect the numerous interrelated programmatic and methodological challenges in delivering services to, and conducting research about, a population defined by its temporal and spatial movements [19, 38, 129]. One of the principal challenges we observed lay in the lack of a clear operational definition of nomadic pastoralism given the range in its expression along the continuum from nomadic to sedentary, also noted by Randall in her analysis of demographic methods that systematically exclude nomadic groups from censuses and maps [19]. Furthermore, in order to enumerate or sample a population, one must first locate that population [16]. This is perhaps one reason for the scarcity of census data on nomadic pastoralist and other mobile groups across the globe.

Another methodological issue relates to the challenge of separating common tropes from the evidence base, as many authors (including those of this review) are likely “outsiders” whose etic viewpoint may shape hypotheses and data interpretation. Stereotypes about nomadic pastoralists, for example, as “ignorant” and “uneducated” or as “wanderers”–which implies aimlessness–ignore the realities of a modern dual lifeway that both observes centuries of tradition while availing itself of 21^st^ century technology. These stereotypes ignore the role that globalization has played in the adoption of contextually appropriate technologies that link them to non-nomadic communities, ideas and economic markets. Despite lifestyle differences that distinguish nomadic pastoralists from their sedentary pastoralist counterparts, utilization of mobile banking, text-messaging, global-positioning systems, and the internet (where available) have increased the number and strength of nomadic pastoralists’ cultural and economic ties, however, explicit recognition of this duality was largely unrecognized in much of the biomedical literature we reviewed [19]. A wider, more inclusive research perspective requires surmounting the barriers of culture, power dynamics and language to engage meaningfully with the nomadic pastoralists as collaborators as well as subjects. Transdisciplinary research methods such as co-design and governance of research, engagement of community members in conduct of research, key informant interviews, public meetings, and community-based participatory techniques such as pair-wise ranking, pile sorting, or free listing, can be effective means to elicit and engage an entire community [93, 130]. Although there are limited resources about community-based participatory data collection techniques in nomadic populations, the more established literature base for refugees and for transdisciplinary research more generally offers approaches that could be adapted [131–133].

A number of papers drew conclusions or made recommendations that were not substantiated by the empirical data from their studies. For example, few studies reporting distance as a barrier to health services uptake included an empirical measure of distance to the health service in question, addressed its seasonal relevance, or compared this distance to other services and resources that nomadic pastoralists routinely access (e.g., markets, water points) to determine if distance *per se* was the critical barrier or if this was a proxy for other barriers (e.g., the direct and indirect costs of seeking care). Correlation between distance and health-seeking or uptake was in several cases presented as causality, even when researchers did not directly inquire about the role of distance, or failed to triangulate the reports alleging distance as a barrier against other competing priorities (i.e., bringing perishable agricultural products to market, or watering their animals). Some studies based their research hypothesis on previously published reports that also did not measure actual distances. This practice reinforces preconceived notions about “reachability” and access, similarly explored in the context of malaria by Smith and Whittaker [129]. Kruger challenges the sole impact of distance, noting that pastoralists’ seeking of services in a particular locality “may not be a linear function of distance,” an assumption we encountered in many papers, but rather a function of “perceived quality of care,” which can impact decision-making either in favor of, or against, formal health services. Similarly, of the 23 studies that described financial barriers, only seven included any empirical measure of direct costs of care (e.g., treatment fees, transport to the health center) or indirect costs of time spent seeking care. The nomadic lifestyle further challenges the applicability of conventional measurement tools such as wealth indices that are typically designed with sedentary populations in mind and focus on infrastructure (e.g., housing type, water sources, utilities) and household goods (e.g., TVs, bicycles) that bear little relevance to a nomadic lifestyle. As noted earlier, pastoralism is an adaptation to changing environmental conditions and thus economic status, like residence, is dynamic and not easily categorized. Our results reinforce previous calls for more holistic paradigms of health care uptake that contextualize both service delivery and health behaviors relative to livelihood assets and their mobilization, and that consider not only vulnerability but resilience [134, 135].

### Research paradigms

Our review also highlighted the tensions between supply-side versus demand-side paradigms in understanding how to improve health services for nomadic pastoralists. We observed that these paradigms were further differentiated by researchers into those that expressed more intrinsic values of improving the well-being and health equity of this often-marginalized population to more extrinsic, instrumental values of reaching nomadic pastoralists for programmatic purposes (e.g., to control infectious diseases in the broader population). As shown in Table 3, the intersection of supply- versus demand-side paradigms and intrinsic versus instrumental value creates four broad research paradigm categories. In general, researchers who focused on the intrinsic value of health service uptake tended to cede the locus of agency to the nomadic pastoralists themselves, encouraging participatory research and community-driven solutions.

**Table 3:** Categories of research paradigms identified.

	Intrinsic Value	Instrumental value
**Supply-side**	We [outside “experts”] need to increase health services uptake among nomadic pastoralists for their own good”. Though external agents set the programmatic priorities, nomadic pastoralists can shape the terms of engagement.	“We [outside “experts”] need to increase health services uptake among nomadic pastoralists for the benefit of the broader population.” Health service delivery to nomadic pastoralists is a means to a given end designed and prioritized by external agents.
**Demand-side**	Nomadic pastoralists define their own priorities and needs which are treated as valid, and their inclusion and ownership are essential. Control of conditions that impact services should be devolved to them so they can achieve and sustain their self-defined ‘good’.	Creating demand in nomadic pastoralists is a necessary good for the entire population. Adoption of services is a matter of understanding and shaping nomadic pastoralist demand to accomplish broader programmatic ends.

Supply-side paradigms emphasized solutions focused on what the health service provider could do to better reach nomadic pastoralists with medical services. These included strategies such as mobile outreach, integration of human health and animal vaccination services, training new cadres of CHWs from nomadic pastoralist communities, educating nomadic pastoralist communities about diseases and available health services, and improving service quality and reception of nomadic pastoralists at fixed-post health centers. Multiple studies focusing on supply-side factors featured implicit assumptions that the health services being offered were appropriate and helpful and should be desirable to nomadic pastoralists (i.e., intrinsically “good”), whereas only a few papers recognized and mentioned the potential repercussions of ineffective care or adverse events associated with poor medical care.

Demand-side paradigms that considered nomadic pastoralists as autonomous agents with legitimate choices, preferences, and constraints, and which highlighted reasons why they might choose to forego formal health services, were less common. Recommendations to improve uptake that were rooted in a demand-side paradigm included community-agency models to devolve design and implementation of health interventions to nomadic pastoralist communities themselves, and proposals to increase community involvement in planning and decision-making around health services and other resources at all levels (from national to local). In a number of studies that looked at low utilization of skilled birth attendants, a seemingly complex problem was explained by a clear demand-side preference [91, 123, 136]. When probed, nomadic pastoralists communities expressed a clear preference for traditional birth attendants, regardless of training level, due to their standing and familiarity within the community and that individuals were chosen by the community itself [136].

It is important to consider both paradigms to accurately diagnose reasons for, and to improve, limited uptake, especially as some factors could be viewed as an intersection (or cycle) of supply and demand factors with the potential for intervention from either side. For example, nomadic pastoralists’ reported preference to seek care from traditional healers rather than government health clinics (a demand-side issue) may have been due to the unreliability of services due to personnel shortages, medication and vaccine stockouts, perceived poor-quality treatment received at government health clinics (a supply-side issue) or to the potentially higher costs of care at a government clinic (typically a supply-side issue where clinic fees exist). However, in some papers, this observed preference for traditional healers was not examined further and was instead characterized as an uninformed belief in the virtues of traditional medicine, thus presenting what may have been a supply-side provider problem as a demand-side problem of consumer ignorance. Limited attention was given to how nomadic pastoralists’ livelihood assets (i.e., human, physical, financial, natural, and social capital) mediated the relationship between supply and demand for health services [134].

### Understanding Context

We noted that several biomedical research efforts failed to situate the issues within a broader societal context by not explicitly recognizing or measuring how factors such as cultural, political or armed conflict can limit access. Although some studies noted the minority status of traditionally nomadic groups and touched on conflicts related to access to water and grazing for their livestock, the implications of a majority-dominated health system were largely unaddressed, though symptoms of that hegemony such as language and cultural barriers within the system were noted. Indirect effects of conflict may be more difficult to measure, for example when perceived threats of violence deter patients’ health-seeking behavior or providers’ willingness or logistical ability to deliver services, as during the insurgencies of Boko Haram in northern Nigeria and Al Shabab in Somalia [137–139].

In the literature reviewed, the ‘problem sets’ (i.e., the framing of the problem to be solved) were almost uniformly examined from the perspective of the researcher or service provider. However, some papers inverted the role of mobility from constraint to advantage. For example, Hampshire captured a critical aspect of context related to gender and age-related norms [42]. Although both can be socially constraining in specific settings, e.g., for junior wives in polygamous households, mobility offers women an opportunity to capitalize on the informal and temporary peer networks that emerge, coalesce and re-group during the course of migrations. Other authors have also referenced successful disease control strategies whereby services were delivered in contexts (such as wells, watering holes and markets) that did not present economic opportunity costs to the nomadic pastoralists [56, 129, 140]. Considering multiple perspectives within and outside of nomadic pastoralists communities is therefore important when defining health threats and opportunities, as factors that may be considered risks from one perspective may contribute to resilience from another [135].

The body of “One Health” research makes a compelling case for the appropriateness and robustness of service delivery models that consider human health in an ecological context by offering integrated care for pastoralists and their herds. These can be contrasted with biomedical approaches that prioritize human health without recognition of the pastoralists’ own prioritization of herd health, sometimes at the expense of human needs [35, 39, 61, 67, 93, 141–143]. “One Health” models recognize that herd health, for cultural and economic reasons, can be a higher priority to the community than an individual human’s and that “provider-centric” service models impart opportunity costs with real economic consequences for nomadic pastoralists. These researchers note that even in the absence of formal integrated, cross-Ministry efforts, collaborating with trusted veterinary staff to design and implement health services can enhance programmatic effectiveness [49, 94].

### Representativeness of the Evidence Base

We observed that the distribution of the literature does not reflect the burden of disease specific to the region. According to the World Health Organization (WHO), all-age mortality in Africa over the 2000–2016 period is attributed (in descending order of top causes) to lower respiratory infections (LRI), HIV/AIDS, diarrheal diseases, non-communicable cardiovascular (stroke and ischemic heart disease), birth-related conditions, and road injuries, and among children under 5, LRI, birth-related conditions, diarrhea and malaria predominate [144]. Yet little research focused on diarrhea or acute respiratory infections, whereas papers related to eradication (e.g., polio) and some elimination (e.g., TB) initiatives were overrepresented relative to their estimated disease burden. These patterns may possibly reflect funding opportunities related to increased global efforts for these diseases [145].

We also noted that the literature was not geographically representative of the region and specifically countries known for nomadic pastoralism. The African Union (AU) considers almost all member countries as “pastoralist” in some form, and while nomadic pastoralism is not practiced universally in Africa, only 29% of AU countries are represented among the papers meeting our inclusion criteria [18]. Research meeting our criteria from countries with known nomadic populations such as Eritrea, Djibouti, Cameroon, Mauritania, Algeria, Egypt, Morocco, and Libya were not identified through our review process. Similarly, twenty-one (38%) of AU member countries list French as an official language, however only five French-language papers meeting our inclusion criteria were identified, three of which focused on francophone countries. To some degree, this may reflect the overall dominance of the English language in scientific publishing [146].

### Limitations

There are limitations to our review. In the absence of standardized terminology regarding nomadic pastoralism, we sought to cast a wide net using terms characterizing or synonymous with our study subject; however, there is the possibility that papers including nomadic populations as a subject or subset may have been excluded if they did not feature the keywords and MeSH terms used. Given the focus of the AU Policy Framework for Pastoralism in Africa, we restricted our search to all continental AU members, thereby excluding seven island nations [18]. In order to further narrow results from the initial search resulting in 5647 papers, we focused on peer-reviewed, original research that clearly identified the target population in the abstract or title (833), potentially excluding relevant sources. By limiting our search to peer-reviewed research articles, we may have omitted findings from reports, abstracts, proceedings, books, book chapters, or other grey literature. Although the inclusion criteria were explicit, evaluating abstracts required some subjective judgement by the reviewers and may have resulted in the loss of some relevant papers. Similarly, while the reviewers sought explicit designations of barriers, facilitators and programmatic recommendations in the papers, individual reviewers may have interpreted imprecise language differently.

### Conclusions and Recommendations

The essential nature of nomadism presents a conundrum to deliver health services effectively and conduct operational research about their uptake. While we intentionally examined gaps and best practices for research from supply- and demand side perspectives, we did not anticipate the results would lend themselves to further parsing into the “unspoken” lens of researchers’ values. However, the way in which researcher values were clearly, if unintentionally expressed, reinforced the objective findings related to research area, approaches and topics.

Given the breadth of African nomadic pastoralist cultures and contexts, health-related services, and diseases included in this systematic review, we focus our recommendations on how to strengthen operational research about health service uptake in this population, as programmatic recommendations will necessarily be context-specific (Table 4).

**Table 4:** Key recommendations for future research on nomadic pastoralists populations.

**1. Use clear operational definitions and measure key constructs:** Future research should focus on a well-defined operational definition of the research subjects grounded in a country- and/or ethnic-group specific literature review. Explicit recognition of the heterogeneity with which nomadic pastoralism is practiced, and how characteristics manifest in a specific context (e.g., shifts in responsibilities by age and gender), will inform study design and considerations such as seasonality [147]. Well-articulated operational definitions of, and means to measure, hypothesized barriers and facilitators are also of paramount importance. Care should be taken by researchers, reviewers, and editors to ensure that conclusions and recommendations are rooted in empirical data and not merely echoing conventional wisdom and common tropes.
**2. Leverage new technologies:** We encourage future investigators to avail themselves of new technologies such as smartphone metadata, biometric data, and automated cellular communications capabilities (SMS, interactive-voice response) for use in the field, and especially to collect geospatial information to strengthen overall data quality [13, 14, 16, 67, 148]. We observed that these technologies were underutilized despite the extraordinary rise of mobile phone and internet coverage and valuable insights that spatial analyses can provide. Many free open-source data collection tools such as Open Data Kit^©^ can function offline, and upload to a cloud server whenever internet connectivity is regained, thereby offering significant potential for expanded data (and meta-data) collection. Additionally, while grazing areas sought by nomadic pastoralists are typically remote and often “off the grid,” this does not preclude the collection of geospatial data that can be collected offline as it not reliant on cellular communications infrastructure [148]. The location of seasonal and temporary settlements, and the distances to waterholes, grazing areas, markets, and health centers, can produce critical data to inform policy, planning and delivery of health services.
**3. Conduct participatory research:** Given the dearth of research about a very diverse population, we encourage researchers to engage in participatory formative research to inform the development of hypotheses, and to design relevant and valid research instruments. Through meaningful dialogue with research participants, transdisciplinary and participatory approaches also help to minimize etic biases by creating the opportunity for researchers to see nomadic pastoralists as collaborators and agents rather than “subjects,” which ultimately strengthens the evidence base and results in the generation of contextually-specific, policy-relevant findings and recommendations [130, 132].
**4. Theorize causal effects:** Future research should include more careful theorization (such as conceptual maps and causal diagrams) to explicate the cause-and-effect relationships being investigated and the potential for feedback loops between supply- and demand-side factors.
**5. Review grey literature:** A systematic review of the “grey literature” would be of value to reveal a body of data that has been collected but remains in limited circulation.
**6. Expand country settings and diseases studied:** Additional research is needed on African nomadic pastoralist groups in francophone and lusophone country settings and on health conditions that represent a larger share of the burden of disease within the region generally and for nomadic pastoralist communities specifically.

Continued operational research in this area could contribute greatly to our understanding of disease dynamics among nomadic pastoralist societies, their valuation of traditional and formal health services, and strategies to improve services in the interest of achieving equity in health for these populations.

## Supporting information

S1 Checklist PRISMA checklist. (DOC)

S1 Fig Flow diagram of article inclusion. (TIF)

S2 Fig Health care research topic and publication year. (TIF)

S1 Appendix Search strategy. (DOCX)
