# Supplementary figures and images for: Health services uptake among nomadic pastoralist populations in Africa: A systematic review of the literature
Source: PLoS Negl Trop Dis. 2020 Jul 27;14(7):e0008474. doi: 10.1371/journal.pntd.0008474 (PMC7447058; doi:10.1371/journal.pntd.0008474)

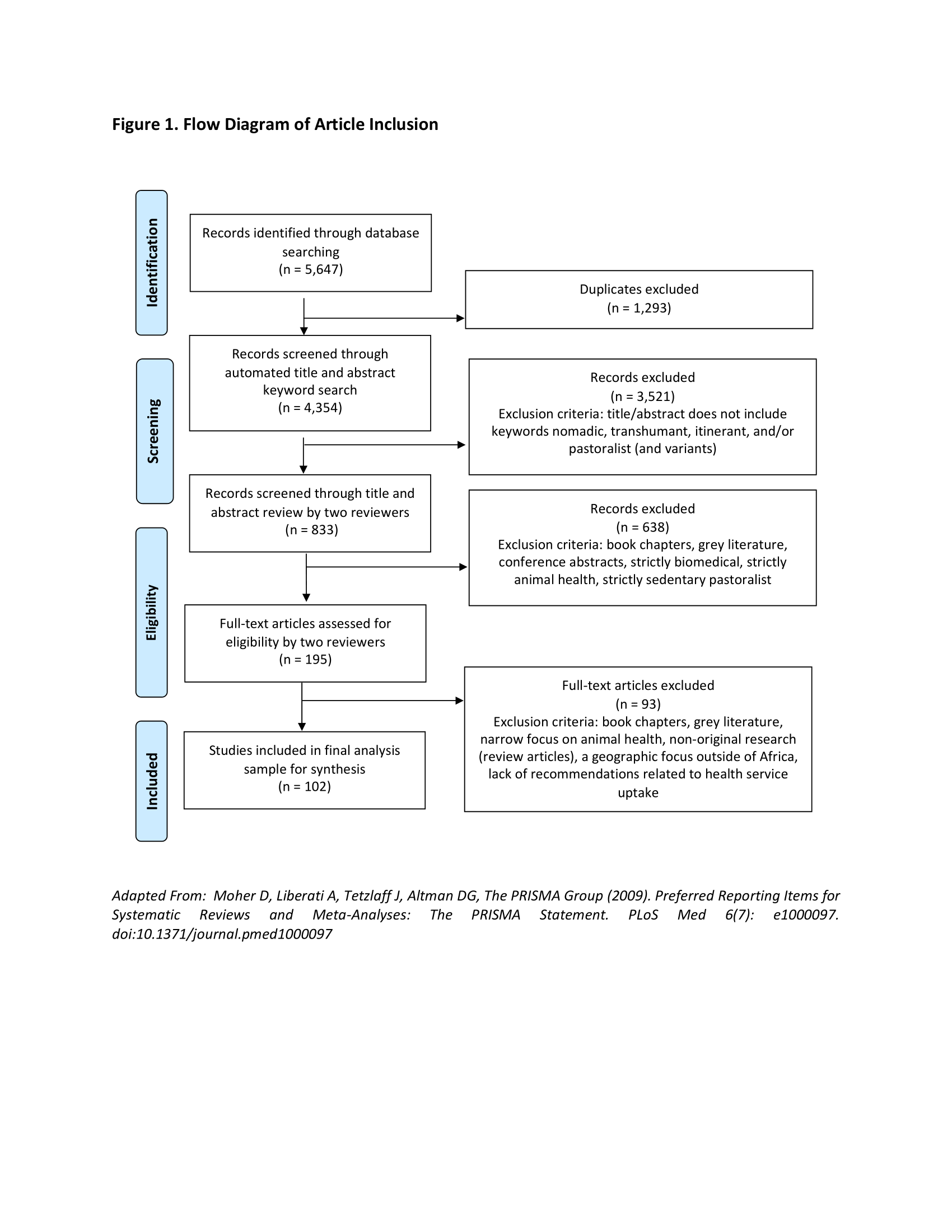

Supplement: S1 Fig — (TIF) [file pntd.0008474.s002.tif]

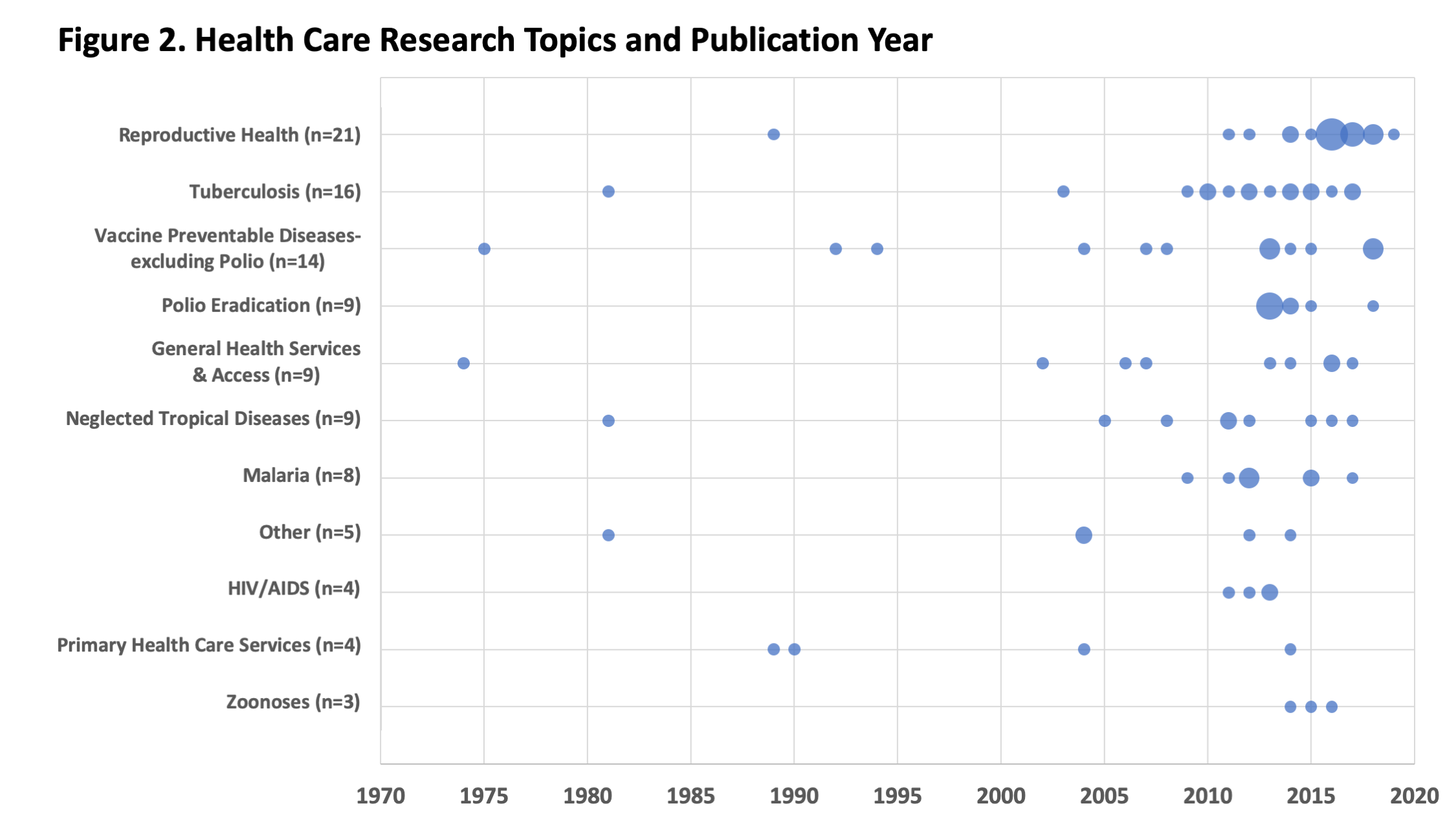

Supplement: S2 Fig — (TIF) [file pntd.0008474.s003.tif]
