# Supplementary material for: Health services uptake among nomadic pastoralist populations in Africa: A systematic review of the literature
Source: PLoS Negl Trop Dis. 2020 Jul 27;14(7):e0008474. doi: 10.1371/journal.pntd.0008474 (PMC7447058; doi:10.1371/journal.pntd.0008474)
Supplement: S1 Appendix — (DOCX) [file pntd.0008474.s004.docx]

**S3 Appendix: Search strategy**

| **Database** | **Strategy** | **Run Date** | **Reference Download** |
| --- | --- | --- | --- |
| **Medline**  **(OVID)**  **1946-** | "Transients and Migrants"/ OR (nomad* OR pastoralist* OR transhuman* OR migrant*).ti,ab.  AND  "Patient Acceptance of Health Care"/ OR Health Promotion/ OR Health care/ OR Health Services/ OR "Health Services Needs and Demand"/ OR Health Planning/ OR Health Services Accessibility/ OR Health Behavior/ or Attitude to Health/ or Health Knowledge, Attitudes, Practice/ OR Disease Outbreaks/ OR exp Vaccination/ OR exp immunization/ OR (health* OR vaccin* OR immuni?ation*).ti,ab.  AND  Exp Africa/ OR (Africa* OR Algeria OR Egypt OR Libya OR Morocco OR Tunisia OR Sub-Sahara* OR Cameroon OR Chad OR Congo OR Guinea OR Gabon OR Burundi or Djibouti or Eritrea or Ethiopia or Kenya or Rwanda or Somalia or Sudan or Tanzania or Uganda or Angola or Botswana or Lesotho or Malawi or Mozambique or Namibia or South Africa or Swaziland or Zambia or Zimbabwe or Benin or Burkina Faso or Cape Verde or Cote d'Ivoire or Gambia or Ghana or Guinea or Guinea-Bissau or Liberia or Mali or Mauritania or Niger or Nigeria or Senegal or "Sierra Leone" or Togo).ti,ab. | 2/17/2016 | 1252  Duplicates Removed = 1250 |
| **Embase**  **(OVID)**  **1947-** | Migration/ OR (nomad* OR pastoralist* OR transhuman* OR migrant*).ti,ab.  AND  "Patient Attitude"/ OR Health Promotion/ OR Health/ OR health care/ OR Health Service/ OR Health care planning/ OR Health care delivery/ OR health care access/ OR Health Behavior/ OR Attitude to Health/ OR epidemic/ OR exp Vaccination/ OR exp immunization/ OR (health* OR vaccin* OR immuni?ation*).ti,ab.  AND  Exp Africa/ OR (Africa* OR Algeria OR Egypt OR Libya OR Morocco OR Tunisia OR Sub-Sahara* OR Cameroon OR Chad OR Congo OR Guinea OR Gabon OR Burundi or Djibouti or Eritrea or Ethiopia or Kenya or Rwanda or Somalia or Sudan or Tanzania or Uganda or Angola or Botswana or Lesotho or Malawi or Mozambique or Namibia or South Africa or Swaziland or Zambia or Zimbabwe or Benin or Burkina Faso or Cape Verde or Cote d'Ivoire or Gambia or Ghana or Guinea or Guinea-Bissau or Liberia or Mali or Mauritania or Niger or Nigeria or Senegal or "Sierra Leone" or Togo).ti,ab. | 2/17/2016 | 1907  Duplicates Removed =1242 |
| **Global Health**  **(OVID)**  **1910-** | Migrants/ OR (nomad* OR pastoralist* OR transhuman* OR migrant*).ti,ab.  AND  Health Promotion/ OR Health care/ OR Health Services/ OR "Health Services Needs and Demand"/ OR Health Planning/ OR Health Services Accessibility/ OR Health Behavior/ or Attitude to Health/ or Health Knowledge, Attitudes, Practice/ OR Disease Outbreaks/ OR exp Vaccination/ OR exp immunization/ OR (health* OR vaccin* OR immunization*).ti,ab.  AND  Exp Africa/ OR (Africa* OR Algeria OR Egypt OR Libya OR Morocco OR Tunisia OR Sub-Sahara* OR Cameroon OR Chad OR Congo OR Guinea OR Gabon OR Burundi or Djibouti or Eritrea or Ethiopia or Kenya or Rwanda or Somalia or Sudan or Tanzania or Uganda or Angola or Botswana or Lesotho or Malawi or Mozambique or Namibia or South Africa or Swaziland or Zambia or Zimbabwe or Benin or Burkina Faso or Cape Verde or Cote d'Ivoire or Gambia or Ghana or Guinea or Guinea-Bissau or Liberia or Mali or Mauritania or Niger or Nigeria or Senegal or "Sierra Leone" or Togo).ti,ab. | 2/17/2016 | 940  Duplicates Removed =621 |
| **PsycInfo**  **(OVID)**  **1806-** | Human Migration/ OR (nomad* OR pastoralist* OR transhuman* OR migrant*).ti,ab.  AND  Health Care Delivery/ OR Health Promotion/ OR Health care utilization/ OR Health Care Services/ OR Health Service Needs/ OR Health Behavior/ OR Health Attitudes/ OR Health Knowledge/ OR Disease Outbreaks/ OR exp Vaccination/ OR exp immunization/ OR (health* OR vaccin* OR immuni?ation*).ti,ab.  AND  (Africa* OR Algeria OR Egypt OR Libya OR Morocco OR Tunisia OR Sub-Sahara* OR Cameroon OR Chad OR Congo OR Guinea OR Gabon OR Burundi or Djibouti or Eritrea or Ethiopia or Kenya or Rwanda or Somalia or Sudan or Tanzania or Uganda or Angola or Botswana or Lesotho or Malawi or Mozambique or Namibia or South Africa or Swaziland or Zambia or Zimbabwe or Benin or Burkina Faso or Cape Verde or Cote d'Ivoire or Gambia or Ghana or Guinea or Guinea-Bissau or Liberia or Mali or Mauritania or Niger or Nigeria or Senegal or "Sierra Leone" or Togo).ti,ab. | 2/17/2016 | 273  Duplicates Removed =152 |
| **Sociological Abstracts**  **(ProQuest Central)** | TI,AB(nomad* OR pastoralist* OR transhuman* OR migrant*)  AND  TI,AB("health care" OR healthcare OR "health service*" OR "health promotion" OR "health behavior" OR "health seeking behavior" OR "health attitude" OR "attitude to health" OR "health knowledge" OR "health needs" OR "disease outbreak*" OR immunization* OR vaccination* OR vaccine*)  AND  TI,AB(Africa OR Algeria OR Egypt OR Libya OR Morocco OR Tunisia OR Sub-Sahara* OR Cameroon OR Chad OR Congo OR Guinea OR Gabon OR Burundi or Djibouti or Eritrea or Ethiopia or Kenya or Rwanda or Somalia or Sudan or Tanzania or Uganda or Angola or Botswana or Lesotho or Malawi or Mozambique or Namibia or South Africa or Swaziland or Zambia or Zimbabwe or Benin or Burkina Faso or Cape Verde or Cote d'Ivoire or Gambia or Ghana or Guinea or Guinea-Bissau or Liberia or Mali or Mauritania or Niger or Nigeria or Senegal or "Sierra Leone" or Togo) | 2/17/2016 | 53  Duplicates Removed =30 |
| **Scopus** | (nomad* OR pastoralist* OR transhuman* OR migrant*)  AND  ("health care" OR healthcare OR "health service*" OR "health promotion" OR "health behavior" OR "health seeking behavior" OR "health attitude" OR "attitude to health" OR "health knowledge" OR "health needs" OR "disease outbreak*" OR immunization* OR vaccination* OR vaccine*)  AND  (Africa* OR Algeria OR Egypt OR Libya OR Morocco OR Tunisia OR Sub-Sahara* OR Cameroon OR Chad OR Congo OR Guinea OR Gabon OR Burundi or Djibouti or Eritrea or Ethiopia or Kenya or Rwanda or Somalia or Sudan or Tanzania or Uganda or Angola or Botswana or Lesotho or Malawi or Mozambique or Namibia or South Africa or Swaziland or Zambia or Zimbabwe or Benin or Burkina Faso or Cape Verde or Cote d'Ivoire or Gambia or Ghana or Guinea or Guinea-Bissau or Liberia or Mali or Mauritania or Niger or Nigeria or Senegal or "Sierra Leone" or Togo) | 2/17/2016 | 183  Duplicates Removed =166 |
| **African Index Medicus** | (nomad* OR pastoralist* OR transhuman* OR migrant*) | 2/17/2016 | 0 |

Update from 2/17/2016 to 2/28/2019

| **Medline**  **(OVID)**  **1946-** | "Transients and Migrants"/ OR (nomad* OR pastoralist* OR transhuman* OR migrant*).ti,ab.  AND  "Patient Acceptance of Health Care"/ OR Health Promotion/ OR Health care/ OR Health Services/ OR "Health Services Needs and Demand"/ OR Health Planning/ OR Health Services Accessibility/ OR Health Behavior/ or Attitude to Health/ or Health Knowledge, Attitudes, Practice/ OR Disease Outbreaks/ OR exp Vaccination/ OR exp immunization/ OR (health* OR vaccin* OR immuni?ation*).ti,ab.  AND  Exp Africa/ OR (Africa* OR Algeria OR Egypt OR Libya OR Morocco OR Tunisia OR Sub-Sahara* OR Cameroon OR Chad OR Congo OR Guinea OR Gabon OR Burundi or Djibouti or Eritrea or Ethiopia or Kenya or Rwanda or Somalia or Sudan or Tanzania or Uganda or Angola or Botswana or Lesotho or Malawi or Mozambique or Namibia or South Africa or Swaziland or Zambia or Zimbabwe or Benin or Burkina Faso or Cape Verde or Cote d'Ivoire or Gambia or Ghana or Guinea or Guinea-Bissau or Liberia or Mali or Mauritania or Niger or Nigeria or Senegal or "Sierra Leone" or Togo).ti,ab. | 2/28/2019 | 471 |
| --- | --- | --- | --- |
| **Embase**  **(OVID)**  **1947-** | Migration/ OR (nomad* OR pastoralist* OR transhuman* OR migrant*).ti,ab.  AND  "Patient Attitude"/ OR Health Promotion/ OR Health/ OR health care/ OR Health Service/ OR Health care planning/ OR Health care delivery/ OR health care access/ OR Health Behavior/ OR Attitude to Health/ OR epidemic/ OR exp Vaccination/ OR exp immunization/ OR (health* OR vaccin* OR immuni?ation*).ti,ab.  AND  Exp Africa/ OR (Africa* OR Algeria OR Egypt OR Libya OR Morocco OR Tunisia OR Sub-Sahara* OR Cameroon OR Chad OR Congo OR Guinea OR Gabon OR Burundi or Djibouti or Eritrea or Ethiopia or Kenya or Rwanda or Somalia or Sudan or Tanzania or Uganda or Angola or Botswana or Lesotho or Malawi or Mozambique or Namibia or South Africa or Swaziland or Zambia or Zimbabwe or Benin or Burkina Faso or Cape Verde or Cote d'Ivoire or Gambia or Ghana or Guinea or Guinea-Bissau or Liberia or Mali or Mauritania or Niger or Nigeria or Senegal or "Sierra Leone" or Togo).ti,ab. | 2/28/2019 | 378 |
| **Global Health**  **(OVID)**  **1910-** | Migrants/ OR (nomad* OR pastoralist* OR transhuman* OR migrant*).ti,ab.  AND  Health Promotion/ OR Health care/ OR Health Services/ OR "Health Services Needs and Demand"/ OR Health Planning/ OR Health Services Accessibility/ OR Health Behavior/ or Attitude to Health/ or Health Knowledge, Attitudes, Practice/ OR Disease Outbreaks/ OR exp Vaccination/ OR exp immunization/ OR (health* OR vaccin* OR immunization*).ti,ab.  AND  Exp Africa/ OR (Africa* OR Algeria OR Egypt OR Libya OR Morocco OR Tunisia OR Sub-Sahara* OR Cameroon OR Chad OR Congo OR Guinea OR Gabon OR Burundi or Djibouti or Eritrea or Ethiopia or Kenya or Rwanda or Somalia or Sudan or Tanzania or Uganda or Angola or Botswana or Lesotho or Malawi or Mozambique or Namibia or South Africa or Swaziland or Zambia or Zimbabwe or Benin or Burkina Faso or Cape Verde or Cote d'Ivoire or Gambia or Ghana or Guinea or Guinea-Bissau or Liberia or Mali or Mauritania or Niger or Nigeria or Senegal or "Sierra Leone" or Togo).ti,ab. | 2/28/2019 | 139 |
| **PsycInfo**  **(OVID)**  **1806-** | Human Migration/ OR (nomad* OR pastoralist* OR transhuman* OR migrant*).ti,ab.  AND  Health Care Delivery/ OR Health Promotion/ OR Health care utilization/ OR Health Care Services/ OR Health Service Needs/ OR Health Behavior/ OR Health Attitudes/ OR Health Knowledge/ OR Disease Outbreaks/ OR exp Vaccination/ OR exp immunization/ OR (health* OR vaccin* OR immuni?ation*).ti,ab.  AND  (Africa* OR Algeria OR Egypt OR Libya OR Morocco OR Tunisia OR Sub-Sahara* OR Cameroon OR Chad OR Congo OR Guinea OR Gabon OR Burundi or Djibouti or Eritrea or Ethiopia or Kenya or Rwanda or Somalia or Sudan or Tanzania or Uganda or Angola or Botswana or Lesotho or Malawi or Mozambique or Namibia or South Africa or Swaziland or Zambia or Zimbabwe or Benin or Burkina Faso or Cape Verde or Cote d'Ivoire or Gambia or Ghana or Guinea or Guinea-Bissau or Liberia or Mali or Mauritania or Niger or Nigeria or Senegal or "Sierra Leone" or Togo).ti,ab. | 2/28/2019 | 51 |
| **Sociological Abstracts**  **(ProQuest Central)** | TI,AB(nomad* OR pastoralist* OR transhuman* OR migrant*)  AND  TI,AB("health care" OR healthcare OR "health service*" OR "health promotion" OR "health behavior" OR "health seeking behavior" OR "health attitude" OR "attitude to health" OR "health knowledge" OR "health needs" OR "disease outbreak*" OR immunization* OR vaccination* OR vaccine*)  AND  TI,AB(Africa OR Algeria OR Egypt OR Libya OR Morocco OR Tunisia OR Sub-Sahara* OR Cameroon OR Chad OR Congo OR Guinea OR Gabon OR Burundi or Djibouti or Eritrea or Ethiopia or Kenya or Rwanda or Somalia or Sudan or Tanzania or Uganda or Angola or Botswana or Lesotho or Malawi or Mozambique or Namibia or South Africa or Swaziland or Zambia or Zimbabwe or Benin or Burkina Faso or Cape Verde or Cote d'Ivoire or Gambia or Ghana or Guinea or Guinea-Bissau or Liberia or Mali or Mauritania or Niger or Nigeria or Senegal or "Sierra Leone" or Togo) | 2/28/2019 | 0 |
| **Scopus** | (nomad* OR pastoralist* OR transhuman* OR migrant*)  AND  ("health care" OR healthcare OR "health service*" OR "health promotion" OR "health behavior" OR "health seeking behavior" OR "health attitude" OR "attitude to health" OR "health knowledge" OR "health needs" OR "disease outbreak*" OR immunization* OR vaccination* OR vaccine*)  AND  (Africa* OR Algeria OR Egypt OR Libya OR Morocco OR Tunisia OR Sub-Sahara* OR Cameroon OR Chad OR Congo OR Guinea OR Gabon OR Burundi or Djibouti or Eritrea or Ethiopia or Kenya or Rwanda or Somalia or Sudan or Tanzania or Uganda or Angola or Botswana or Lesotho or Malawi or Mozambique or Namibia or South Africa or Swaziland or Zambia or Zimbabwe or Benin or Burkina Faso or Cape Verde or Cote d'Ivoire or Gambia or Ghana or Guinea or Guinea-Bissau or Liberia or Mali or Mauritania or Niger or Nigeria or Senegal or "Sierra Leone" or Togo) | 2/28/2019 | 0 |
| **African Index Medicus** | (nomad* OR pastoralist* OR transhuman* OR migrant*) | 2/28/2019 | 0 |
